# Identification of Clonal Neoantigens Derived From Driver Mutations in an *EGFR*-Mutated Lung Cancer Patient Benefitting From Anti-PD-1

**DOI:** 10.3389/fimmu.2020.01366

**Published:** 2020-07-23

**Authors:** Di Wu, Yangyang Liu, Xiaoting Li, Yiying Liu, Qifan Yang, Yuting Liu, Jingjing Wu, Chen Tian, Yulan Zeng, Zhikun Zhao, Yajie Xiao, Feifei Gu, Kai Zhang, Yue Hu, Li Liu

**Affiliations:** ^1^Cancer Center, Union Hospital, Tongji Medical College, Huazhong University of Science and Technology, Wuhan, China; ^2^YuceBio Technology Co., Ltd., Shenzhen, China

**Keywords:** neoantigens, cancer immunotherapy, immune checkpoint blockade, epidermal growth factor receptor, tyrosine kinase inhibitor

## Abstract

Epidermal growth factor receptor (*EGFR*) tyrosine kinase inhibitors (TKIs) have been recommended as the first-line therapy for non-small cell lung cancer (NSCLC) patients harboring *EGFR* mutations. However, acquired resistance to EGFR-TKIs is inevitable. Although immune checkpoint blockades (ICBs) targeting the programmed cell death 1 (PD-1)/PD-ligand (L)1 axis have achieved clinical success for many cancer types, the clinical efficacy of anti-PD-1/PD-L1 blockades in *EGFR* mutated NSCLC patients has been demonstrated to be lower than those without *EGFR* mutations. Here, we reported an advanced NSCLC patient with *EGFR* driver mutations benefitting from anti-PD-1 blockade therapy after acquiring resistance to EGFR-TKI. We characterized the mutational landscape of the patient with next-generation sequencing (NGS) and successfully identified specific T-cell responses to clonal neoantigens encoded by *EGFR* exon 19 deletion, *TP53 A116T* and *DENND6B R398Q* mutations. Our findings support the potential application of immune checkpoint blockades in NSCLC patients with acquired resistance to EGFR-TKIs in the context of specific clonal neoantigens with high immunogenicity. Personalized immunomodulatory therapy targeting these neoantigens should be explored for better clinical outcomes in *EGFR* mutated NSCLC patients.

## Introduction

Lung cancer, in which about 80% of cases are identified as non-small cell lung cancer (NSCLC), is regarded as the leading cause of cancer-related death in the world (1). Alterations associated with specific genes, such as epidermal growth factor receptor (*EGFR*) or anaplastic lymphoma kinase (*ALK*), contribute to the development and progression of lung cancer. Relevant targeted therapies directing against these driver gene mutations have achieved successful clinical outcomes (2, 3). The *EGFR* driver mutations are known to be prevalent among Asian NSCLC patients (4). Although *EGFR* tyrosine kinase inhibitors (TKIs) could improve the objective response rate (ORR) and progression-free survival (PFS) of *EGFR* mutated patients, acquired resistance is inevitable and often occurs after 9–14 months of therapy (5). Although the administration of third-generation EGFR-TKIs targeting the *EGFR T790M* mutation, such as Osimertinib, has shown promising outcomes (6), acquired resistance still exists (7). Thus, novel effective treatment strategies remain urgently needed.

Recently, immune checkpoint blockades (ICBs), including anti-programmed cell death-1 (PD-1) and programmed cell death-ligand 1 (PD-L1) blockades, have been demonstrated to robustly enhance anti-tumor immunity in patients with a wide range of cancers, especially with NSCLC (8, 9). Despite the sustained response of ICBs in NSCLC, the clinical efficacy of anti-PD-1/PD-L1 blockades in *EGFR* mutated NSCLC patients has been reported to be moderate compared with those without *EGFR* mutations (10, 11). Moreover, results from several clinical trials indicated that the combination therapy of EGFR-TKIs and ICBs led to a high incidence of treatment-related adverse effects (12). As a result, immune checkpoint blockades have been excluded from daily clinical applications for NSCLC patients with *EGFR* driver mutations. Nevertheless, some *EGFR* mutated lung cancer patients enrolled in clinical trials could still respond to ICB therapy. Therefore, it is necessary to characterize the underlying mechanism and identify prognostic biomarkers for predicting clinical benefits with anti-PD-1/PD-L1 blockade therapy in this specific NSCLC subpopulation.

Unlike tumor-associated antigens (TAA), which are found both in tumor cells and normal tissues, tumor neoantigens are exclusively processed by tumor cells and presented by major histocompatibility complex (MHC) molecules. Individual MHC:peptide complex can be recognized by T-cell receptor with high specificity (13, 14). This mechanism provides promising targets for personalized immunomodulatory therapy such as cancer vaccine and adoptive T-cell transfer therapy (15, 16). Interestingly, previous reports suggested that neoantigens can be served as the targets of highly specific and durable anti-tumor immunity (17, 18), and neoantigen-specific T-cell response can be identified in patients benefitting from ICBs. Neoantigens derived from *EGFR* mutations have been reported in preclinical study (19), but it remains confusing whether *EGFR* driver mutations could generate true neoantigens in suppressive tumor microenvironment (TME).

With the development of next-generation sequencing (NGS) technologies and bioinformatics algorithms, neoantigen can be successfully identified *in silico* in many solid tumors (20). Monitoring neoantigen-specific T-cell response to anti-PD-1/PD-L1 blockades in peripheral blood has become a feasible way to predict the prognosis of cancer patients (13, 21). Nevertheless, only a small amount of neoantigens were identified to be truly immunogenic, and clinical applications based on neoantigens are still in its infancy stage (17). Given the current limiting treatment options for NSCLC patients after EGFR-TKI resistance, novel personalized therapeutic strategies based on T-cell immunity to neoantigens could improve clinical outcomes when candidate neoantigens are available.

Here, we reported an advanced NSCLC patient with *EGFR* driver mutations achieved durable clinical benefits from Nivolumab monotherapy. By conducting whole-exome sequencing (WES), RNA sequencing (RNA-seq), and TCR sequencing, we were able to depict a comprehensive landscape of genomic alterations and predict candidate neoantigens from tumor tissue obtained before the initiation of Nivolumab. We also successfully validated anti-tumor immunity to some high-quality neoantigens *in vitro*, including two derived from *EGFR* driver mutation. These results displayed that immune checkpoint blockades could elicit robust endogenous T-cell response to clonal neoantigens generated from driver mutations. Our findings may provide clinical evidences that ICBs should not be completely excluded from therapy options for NSCLC patients after the failure of EGFR-TKIs. Furthermore, personalized immunomodulatory therapy targeting specific clonal neoantigens should be developed for clinical practice in the future.

## Result

### Case Presentation

**Figure 1:**
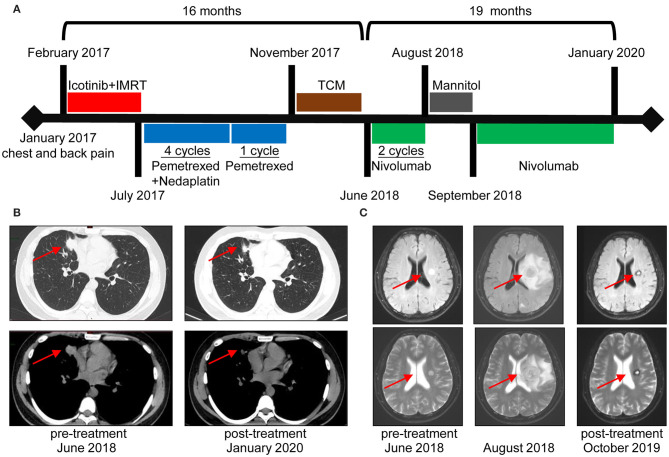
Durable clinical response to Nivolumab in a non-small cell lung cancer (NSCLC) patient with epidermal growth factor receptor (*EGFR*) driver mutations. **(A)** Clinical timeline of patient, with major treatment indicated. The patient has been benefitting from immunotherapy for more than 19 months. **(B)** Chest computed tomography (CT) of the metastatic lung tumors before Nivolumab initiation (June 2018) and last time follow-up (January 2020). **(C)** Magnetic resonance imaging (MRI) before and after Nivolumab treatment. Images in the middle revealed an increased size of the left basal ganglia lesion accompanied by edema and multiple brain nodule metastases. A rapid decrease in lesions was noted in the following radiological evaluations.

A 34-year-old male patient suffered from chest and back pain in January 2017. Radiological examinations revealed a 65-mm nodule in the middle lobe of the right lung, several metastatic pulmonary nodules in both lungs, and multiple bone lesions. The patient underwent a bronchoscopy biopsy, and pathological examination revealed lung adenocarcinoma with *EGFR* exon 19 deletion (*EGFR 19del*). His clinical stage was T4N2M1b stage IV (Figure 1A). The patient was initially treated with Icotinib from February 2017 until progression occurred in July 2017. Additionally, intensity-modulated radiation therapy (IMRT) targeting bone metastases in the lumbar spine, pelvic cavity, and left femur were given with a total dose of 36 Gy in 12 fractions. After that, he was administered with Pemetrexed plus Nedaplatin for four cycles and Pemetrexed for another one cycle until progression occurred in November 2017. After systemic chemotherapy, he turned to traditional Chinese medicine treatment, until the onset of brain metastases in the right frontal lobe and left basal ganglia in June 2018 (Figures 1B,C).

The patient was, thereafter, enrolled in a phase 3 clinical trial for Nivolumab monotherapy (NCT03195491). Regardless of PD-L1 and tumor mutational burden (TMB) status, this trial enrolled advanced lung cancer patients who failed previous systemic therapies. Biopsy of tumor sample obtained before Nivolumab initiation indicated *EGFR T790M* mutation. The patient presented with dizziness after two cycles of Nivolumab administration in August 2018, and magnetic resonance imaging (MRI) scans showed an increased lesion size and edema of the left basal ganglia, as well as multiple brain metastases (Figure 1C). After dehydration therapy with Mannitol, Nivolumab was reinitiated for another two cycles. According to RECIST 1.1 Criteria, he achieved partial response (PR) with decreased tumor size of lung and brain metastases in September 2018. After 10 cycles of Nivolumab treatment, the patient experienced hypothyroidism with elevated levels of TSH and decreased levels of both FT3 and FT4, and treatment with Levothyroxine was applied to relieve the symptoms. Generally, Nivolumab was well-tolerated. Currently, the patient receives an intravenous infusion of Nivolumab at 240 mg every 2 weeks. Based on the follow-up examinations, the patient has achieved PR for more than 1 year.

### Comprehensive Analysis of Genome and Immune Landscape

**Figure 2:**
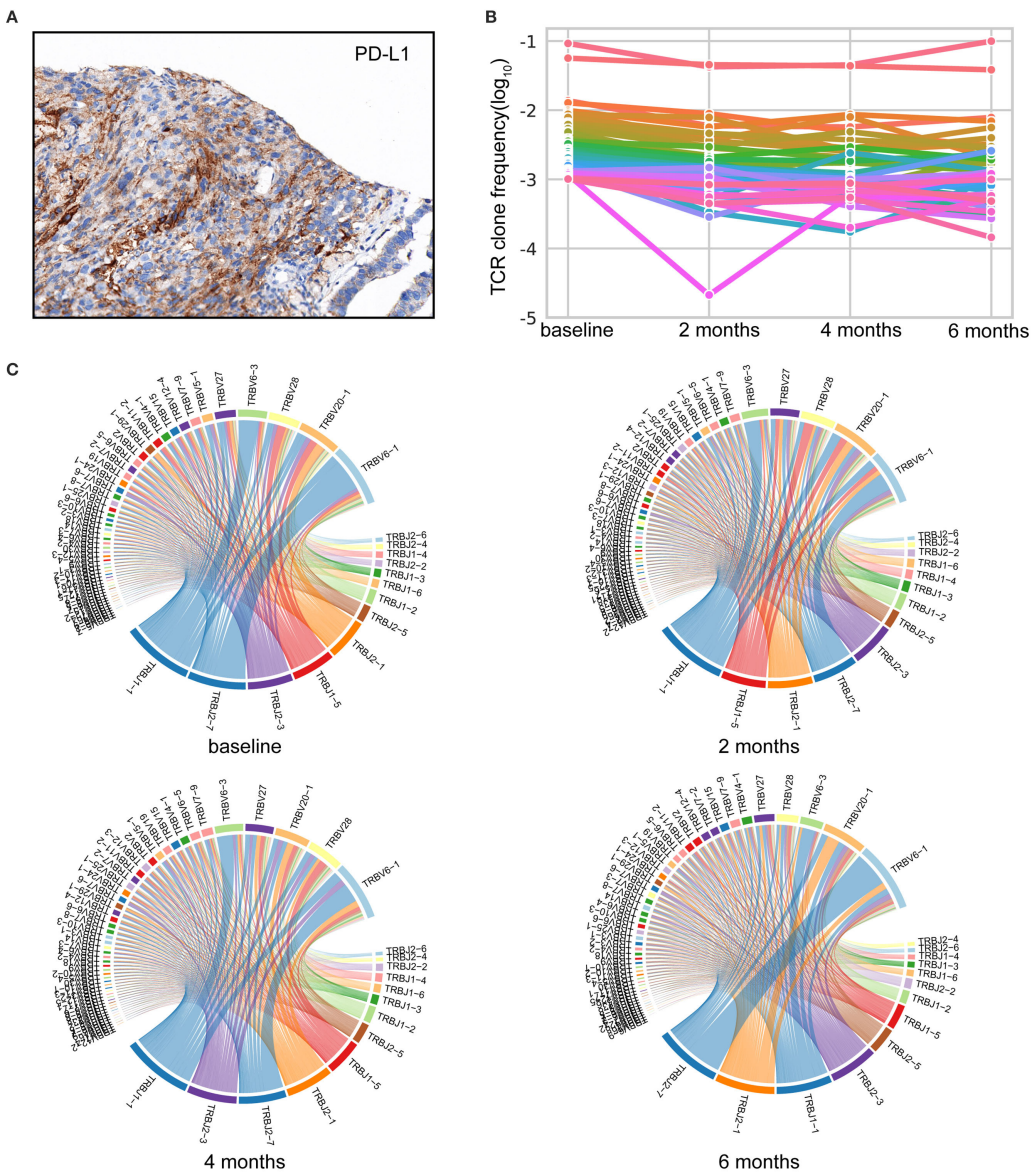
Comprehensive analysis of the immune landscape. **(A)** Immunohistochemistry (IHC) image with anti-programmed cell death-ligand 1 (PD-L1) antibody (Dako IHC 22C3 platform). Microscope magnification 400×. A PD-L1 tumor proportion score (TPS) of ≥50% was detected. **(B)** Maintenance of the high-frequency T-cell clones throughout Nivolumab treatment. TCR-seq was conducted on PBMCs collected pre and post Nivolumab treatment. T-cell clones with a frequency of ≥10^−3^ in the baseline are shown. Each line represents one clone. **(C)** Representative TRBV–TRBJ junction circos plots. Bands represent different V and J gene segments. Ribbons imply V/J pairings. The width of the band is proportional to the usage frequency.

Of the tumor analyzed, a PD-L1 tumor proportion score (TPS) of ≥50% was revealed (Figure 2A). Based on sequence data, tumor mutational burden (TMB) was 6.00 muts/Mb, and tumor neoantigen burden (TNB) was 2.67 neos/Mb. Peripheral blood mononuclear cells (PBMCs) collected before Nivolumab initiation and thereafter every 2 months were investigated by TCR sequencing. We selected T-cell clones with a frequency of ≥10^−3^ to investigate the dynamic TCR changes. The maintenance of most high-frequency clones was detected (Figures 2B,C). Only one high-frequency clone decreased sharply after 2 months of Nivolumab therapy. This may partly explain the pseudoprogression after four cycles of Nivolumab treatment in August 2018 and durable clinical response throughout the whole study.

### Prediction and Validation of Immunogenic Neoantigens

**Figure 3:**
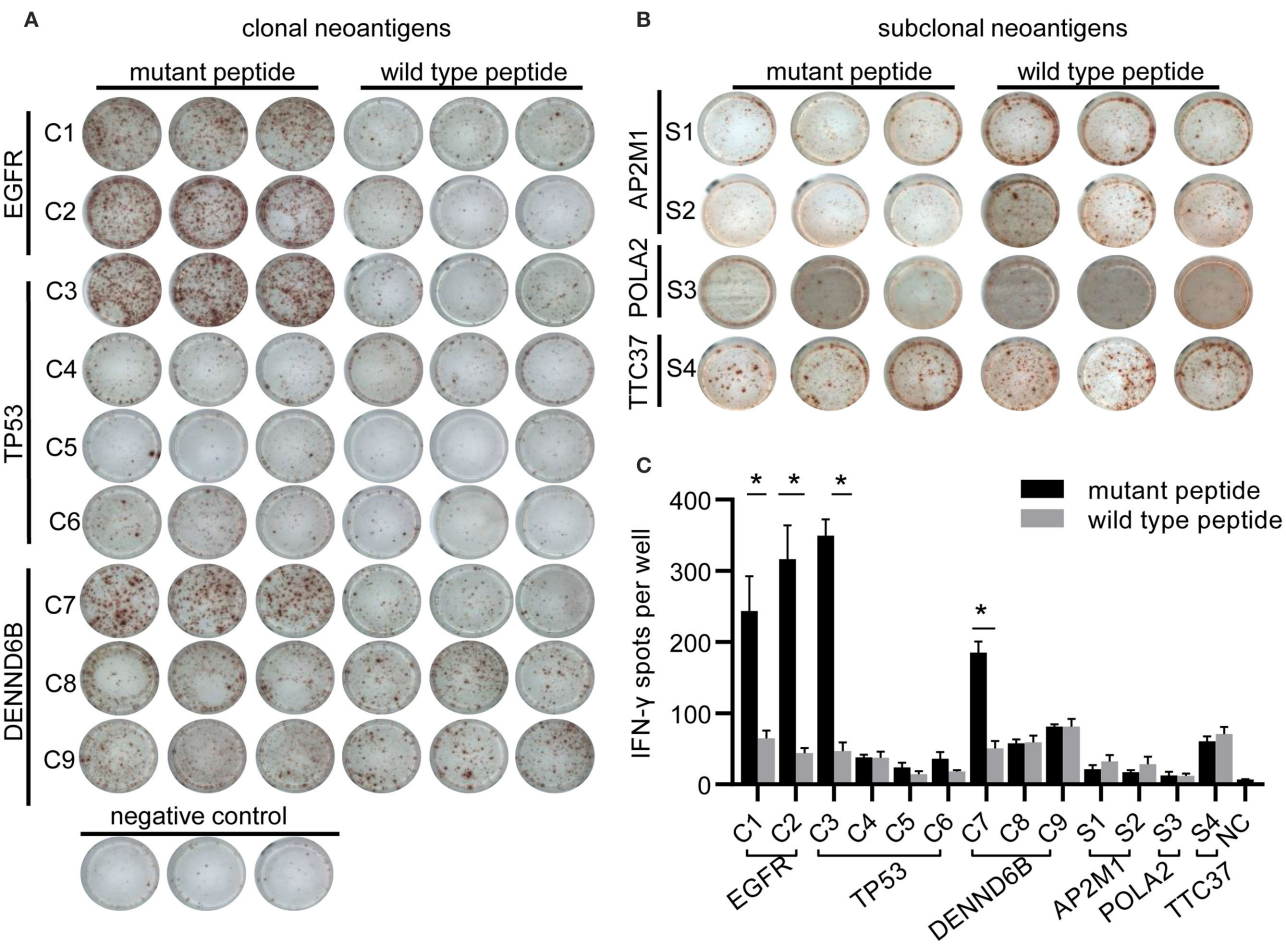
Immunogenicity of candidate neoantigens. **(A,B)** Representative images from IFN-γ ELISPOT assay. Peripheral blood mononuclear cells (PBMCs; 2 × 10^5^ per well) from the patient were stimulated for 10 days with individual peptides in the presence of cytokines (IL-2, IL-7, and IL-15) and were stimulated again on day 10. T-cell reactivity was assessed by IFN-γ ELISPOT assay. **(C)** Bar graph showing IFN-γ ELISPOT assay data. PBMCs stimulated with no peptide were regarded as negative controls (NC). At least two independent experiments were done in triplicate. ^*^p < 0.05.

To evaluate potential factors contributing to the durable response of the patient, we followed a restricted pipeline integrating NGS technology and validation experiment aiming at identifying true neoantigens. According to WES results of lung tumor samples, 84 somatic non-synonymous mutations were identified, and 28 of them were likely to bind to the corresponding HLA alleles with high affinity (IC50 <500 nmol/L) (Supplementary Table 1). A total of six genes were found to be truly expressed at the transcript level, including *EGFR, TP53, POLA2, AP2AM1, DENND6B*, and *TTC37*. Ultimately, 13 HLA-A^*^11:01-restricted candidate neoantigen peptides generated from these six genes were selected for further analysis. Furthermore, clonal neoantigens can be derived from *EGFR, TP53*, and *DENND6B* mutations, whereas *POLA2, AP2AM1*, and *TTC37* mutations could only generate subclonal neoantigens (Table 1). Then, both mutant and wild-type peptides were synthesized and tested by IFN-γ ELISPOT assay to validate the immunogenicity of these neoantigens. As a result, 4 out of 13 mutant peptides could elicit a strong response, whereas the wild-type counterpart generated no significant response. Besides, there were no notable T-cell responses to subclonal neoantigens (Figure 3).

**Table 1:** HLA-A^*^11:01 restricted candidate neoantigens validated in IFN-γ ELISPOT assay.

				**Mutant peptide**		**Wild-type peptide**	
	**Number**	**Gene**	**Mutation**	**Sequence**	IC50 (nM)^*^	**Sequence**	IC50 (nM)^*^
Clonal neoantigens	C1	EGFR	E746_A750del	IPVAIKTSPK	131.9	IPVAIKELRE	28,185.9
	C2	EGFR	E746_A750del	AIKTSPKANK	404.4	AIKELREATS	37,251.5
	C3	TP53	A161T	RVRAMTIYKQ	288.6	RVRAMAIYKQ	486.1
	C4	TP53	A161T	GTRVRAMTIYK	165.5	GTRVRAMAIYK	251.6
	C5	TP53	A161T	TRVRAMTIYK	30.7	TRVRAMAIYK	44
	C6	TP53	A161T	RVRAMTIYK	16.1	RVRAMAIYK	20.9
	C7	DENND6B	R398Q	QLLKGVQKK	498.5	RLLKGVQKK	165.1
	C8	DENND6B	R398Q	KALLKQLLK	54.8	KALLKRLLK	71.5
	C9	DENND6B	R398Q	KQLLKGVQK	420.6	KRLLKGVQK	17,851.5
Subclonal neoantigens	S1	AP2M1	V377M	KASENAIMWK	51.6	KASENAIVWK	91.8
	S2	AP2M1	V377M	ASENAIMWK	41.6	ASENAIVWK	68
	S3	POLA2	E448K	FSYSDLSRK	47.3	FSYSDLSRE	15,881.9
	S4	TTC37	D95A	KDALPGVYQK	171.6	KDDLPGVYQK	7,311.9

* *HLA-binding affinities for peptides, predicted by NetMHCpan v3.0. Peptides with an IC50 <500 nM can be regarded as major histocompatibility complex (MHC) binders*.

### The Dynamic Change of TCR Repertoire After Neoantigen Stimulation

Although IFN-γ ELISPOT assay could reveal the reactivity between immunogenic neoantigens and autologous T cells, we utilized TCR sequencing to further confirm whether T cell responded to neoantigens. Owing to the limited amounts of PBMCs, we only validated the above 4 out of 13 neoantigens generated from *EGFR 19del, TP53 A161T*, and *DENND6B R398Q* mutations.

After stimulating PBMCs with neoantigens, the frequency of some T-cell clones stimulated by mutant peptides was much higher than those clones stimulated by corresponding wild-type peptides. These clones were defined as significant clones, which may specifically recognize neoantigens (Supplementary Table 2). Matching significant clones to those found in blood samples, 18 clones remained at high frequency during this study, and 7 clones could only be detected after the initiation of Nivolumab (Supplementary Figure 1).

## Discussion

Owing to the limited therapeutic strategies for NSCLC patients acquired resistance to EGFR-TKIs, it may be tempting to begin immune checkpoint blockades therapy for its well tolerance and low toxicity. Some retrospective studies suggest that *EGFR* mutated NSCLC cannot benefit from anti-PD-1/PD-L1 blockade therapies (10, 11). However, long-term follow-up of these clinical trials and some case reports showed that a group of *EGFR* mutated NSCLC patients sustained a durable response. Most studies focused on elucidating the underlying mechanism of the negative clinical outcomes (22–25). Little efforts have been made to stratify a small group of patients with *EGFR* driver mutation, who are likely to benefit from immunotherapy. To our knowledge, this is the first report on assessing the predictive biomarker from the perspective of neoantigens in an *EGFR* mutated NSCLC patient receiving Nivolumab. Our results may provide clinical evidences for the potential application of immune checkpoint blockades in NSCLC patients with acquired resistance to EGFR-TKIs.

PD-L1 expression is employed as a general biomarker for immune checkpoint blockade treatment (26). Previous studies demonstrated that EGFR signaling pathway could intrinsically upregulate tumor PD-L1 expression and contribute to the immune escape of *EGFR* mutated NSCLC (10). Conversely, real-world studies showed a higher expression level of PD-L1 in *EGFR* wild-type NSCLC (27, 28). A recent study manifested that the proportion of PD-L1-positive tumors in patients receiving EGFR-TKIs tended to be increased after EGFR-TKI therapy (29). As a result, these contradictory results cannot fully explain the relationship between EGFR signaling pathway and PD-L1 expression. Our patient had a PD-L1 tumor proportion score (TPS) of ≥50%, which may contribute to his durable response to ICBs. For both *EGFR* mutated and wild-type NSCLC in the ATLANTIC trial, patients with higher tumor PD-L1 expression can achieve a better objective response from Durvalumab treatment (29). However, some patients with high PD-L1 expression failed with immunotherapy unexpectedly (30). The opposite results revealed that PD-L1 expression may not be a reliable predictive biomarker for NSCLC with *EGFR* driver mutations receiving immunotherapy, and new effective biomarkers are still needed.

Currently, tumor mutational burden (TMB) is considered as a positive prognostic factor for ICBs (31). Patients with high TMB could have better objective responses to anti-PD-1/PD-L1 blockades compared with those with low TMB (14). Similarly, tumor neoantigen burden (TNB) describes those mutations at the transcript level or protein level and is supposed as the surrogate of TMB. However, some researches showed that patients with high TNB may still be resistant to ICBs (32). On this account, identifying high-quality neoantigens could optimize the prediction of pre-existing immunity to tumors and boost the effect of ICBs for proper immunosurveillance (33).

We presented a pipeline combining *in silico* and *in vitro* approaches to identify true neoantigens. Neoantigens expressed by a large proportion of tumor cells were defined as clonal neoantigens (34). In contrast, subclonal neoantigens may be generated during tumor evolution, which mediated immune escape and facilitated tumor invasion (35). In this study, we only successfully detected T-cell response to clonal neoantigens in IFN-γ ELISPOT assay, which could partly be explained by the loss of subclonal neoantigens after Nivolumab treatment (36). Here, a total of four clonal neoantigens, including two arising from *EGFR 19del*, 1 from *TP53 A161T*, and 1 from *DENND6B R398Q*, respectively, were validated in this case. Although immunotherapy targeting *EGFR* mutations have been widely discussed (34), there was no research on identifying neoantigens generated from *EGFR* mutations in NSCLC patients. Neoantigens derived from hotspot mutations of *TP53*, the most frequently altered gene across solid tumors, have already been screened for novel therapeutic approaches (35). *TP53 A161T*, which is present in approximate 0.06% of cancer patients (37), remained uncharacterized, and future exploration is warranted. Additionally, the role of *DENND6B* in tumors is largely unknown. Based on our results, we speculated that neoantigens derived from driver mutations facilitated clinical benefit in patients treated with immune checkpoint blockades. Therefore, *EGFR* mutated NSCLC patients could be recommended to choose anti-PD-1/PD-L1 blockades in the presence of clonal neoantigens derived from driver mutations with high immunogenicity.

Previous studies had shown that memory T cells from peripheral blood could respond to neoantigens in tumor tissue (38), and CD8^+^PD-1^+/high^ T-cell subsets were preferentially enriched upon neoantigen stimulation (39, 40). Analyzing features of CD8^+^ T cells seemed to be an alternative choice for monitoring immune response. Our former research had already identified the peripheral blood TCR repertoire of NSCLC patients as a useful prognostic biomarker (41). In this study, we investigated the dynamic change in TCR frequencies and found that the maintenance of high-frequency T-cell clones might be associated with a durable response to Nivolumab. By comparing each T-cell clone after neoantigen peptide stimulation, we identified some neoantigen-specific T-cell clones, which are likely responsible for the recognition of MHC:peptide complex. A recent study has raised the hypothesis of clonal replacement after immunotherapy, and researchers founded that expanded T-cell clones were recruited from blood rather than continuously presented in tumors (42). Consistent with the hypothesis, we observed that some significant clones only expanded after the initiation of Nivolumab. Further research should be conducted to characterize the anti-tumor immunity of these T-cell clones.

Besides tumor-intrinsic factors, tumor microenvironment (TME) will also affect the efficacy of immunotherapy. A newly published research focused on TME found that *EGFR* driver gene alterations, despite its inescapable role in tumor growth, contributed directly to a non-inflamed phenotype, with high regulatory T-cell (Treg) infiltration and low CD8^+^ T-cell infiltration (27). For our patient, Nivolumab was initiated only after multiline therapies, including EGFR-TKIs, chemotherapy, and radiotherapy. Thus, we presumed an enhanced suppressive activity of TME, and we did not conduct a comprehensive analysis on TME.

The clinical implications of our research were profound. A previous study showed that the anti-EGFR antibody titer was characterized as highest in NSCLC patients with *EGFR* exon 19 deletion, suggesting that this mutation is immunogenic and can be expressed at protein level (43). We may infer that the peptide sequences derived from *EGFR* exon 19 deletion (IPVAIKTSPK, AIKTSPKANK) may be a potential therapeutic target of cancer vaccine for NSCLC. Moreover, HLA-A^*^11:01 was the most frequent HLA-A allele in Asians (44). *EGFR 19del* is the most common mutation in *EGFR* mutated NSCLC patients, accounting for ~45% of all cases (43). Consequently, it is reasonable that our results can provide important clinical data for a subgroup of NSCLC patients with both *EGFR* mutations and HLA-A^*^11:01 allele.

There are some expected limitations of our study. At first, TCR profiling was only performed on peripheral blood and cultured PBMCs due to a lack of tumor tissue. A mapping of TCR in tumor and metastatic sites will make the results more convincing. Second, recent research has highlighted the important role of MHC class II-restricted neoantigens in the anti-tumor response (45). In this study, we only focused on MHC-I-restricted neoantigens. Investigations of MHC class II-restricted neoantigens may be carried out in the future with more advanced technologies.

Overall, our data suggested that high-quality neoantigen can be generated from *EGFR* driver mutation. ICBs can be used for advanced NSCLC with acquired resistance to EGFR-TKIs in the context of specific clonal neoantigens with high immunogenicity. Monitoring of neoantigen-specific T-cell response might be beneficial for improving the survival rate of NSCLC patients. Personalized immunomodulatory therapy targeting these neoantigens should be explored for better clinical outcomes. We hope our findings may help pave the way for future researches on *EGFR* mutated NSCLC.

## Materials and Methods

### Patient Samples

Peripheral blood was obtained from the patient before the initiation of Nivolumab and every 2 months after treatment. PBMCs were isolated by Ficoll-Hypaque density centrifugation and were analyzed immediately after isolation. Lung cancer tissue sample was obtained by computed tomography (CT)-guided lung biopsy before Nivolumab treatment.

This study was approved by the Institutional Review Board of Tongji Medical College of Huazhong University of Science and Technology. The patient gave his written informed consent for the collection of blood and tissue samples in accordance with the Declaration of Helsinki.

### Immunohistochemistry

The Dako PD-L1 IHC 22C3 pharmDx assay was used to detect PD-L1 protein expression in formalin-fixed paraffin-embedded (FFPE) tumor tissue slides. A four-tiered grading system was applied to evaluate the proportion of PD-L1 expression in tumor cells: TC0 for negative expression, TC1 for 1–5%, TC2 for 5–50%; TC3 for more than 50%.

### Whole-Exome Sequencing and RNA Sequencing

Whole-exome sequencing was carried out on the FFPE tumor tissue and matched normal samples. Peripheral blood was served as normal sample. Genomic DNAs were from tumor tissue, and blood was, respectively, extracted using the Qiagen DNA FFPE and Qiagen DNA blood mini kit (Qiagen). RNA was extracted from FFPE tumor tissue slides using RNeasy FFPE Kit (Qiagen). Sequencing libraries were constructed using Agilent SureSelect Human All Exon V6 kit (Agilent Technologies, USA), and sequencing procedures were performed on an Illumina HiSeq X-Ten platform with 150-bp paired-end reads. Raw reads were filtered using SOAPnuke (v1.5.6) to remove low-quality reads with unknown bases “N” more than 10%. Clean reads were aligned to the human reference genome (UCSCGRCh37/hg19) with the BWA (v0.7.12) for WES and RSEM (v1.3.0) for RNA sequencing. Somatic single-nucleotide variants (SNVs) and indels were identified using VarScan (v2.4.1) and subsequently filtered by an in-house approach to remove the possible false-positive variants (46). Aligned RNA reads were then analyzed using RSEM (v1.3.0). Tumor purity was evaluated computationally in paired samples using AscatNGS (v3.1.0) (47). Tumor mutational burden (TMB) was determined as the number of non-silent somatic mutations per megabase of exome examined. High and low TMB was determined according to a cut-off value of 10 and 2.5 muts/Mb, respectively. The expression of neoantigens was calculated according to both the variant allele frequency of corresponding mutations and the expression level of genes involved. Tumor neoantigen burden (TNB) was defined as the number of neoantigens per megabase of exome examined, and high TNB was determined according to a cut-off value of 4.5 neos/Mb.

### HLA Typing and Neoantigen Prediction

HLA typing of tumor samples and paired normal samples was assessed from WES results using POLYSOLVER (v1.0) and Bwakit (v0.7.11) (48), and were further used for neoantigen prediction. By using in-house software, all the non-silent mutations were translated into 21-mer peptide sequence centered on mutated amino acid. Then, the 21-mer peptide was used to create 9- to 11-mer peptide via a sliding window approach for predicting the binding affinity of major histocompatibility complex class I (MHC I) proteins and their peptide ligands. NetMHCpan (v3.0) was used to determine the binding strength of mutant peptides to patient-specific HLA alleles (49). The predicted peptides were scored according to the following indexes: strong binding affinity, mutation type, variant allele frequency, proteasomal C terminal cleavage, transporter associated with antigen processing, transporting efficiency, and gene expression. Peptides of score higher than 0 were selected. If selected peptides were generated from the same mutation, it can be only counted as one neoantigen.

### *In vitro* PBMC Expansion

PBMCs were rested according to previous studies (16, 50). Autologous PBMCs (2 × 10^5^ cells per well) were co-cultured with separate peptides derived from candidate neoantigens (10 μg/ml) with RPMI-1640 supplemented with 10% fetal bovine serum, 10 U/ml of penicillin–streptomycin, 2 mmol/L L-glutamine, and 1× non-essential amino acid. Cell culture was conducted for 10 days at 37°C in a 5% CO_2_ atmosphere, and half of the culture media was replaced by fresh culture media containing 100 IU/ml IL-2, 50 ng/ml IL-7, and 50 ng/ml IL-15 on days 3, 5, and 7. Half of the culture media was replaced with fresh media without cytokines on day 9.

### IFN-γ ELISPOT Assay

The frequency of neoantigen-specific T cells after 10 days of coculture was determined by IFN-γ ELISPOT kit (Dakewei, China) (51). Briefly, PBMCs (2 × 10^5^ per well) and peptides (10 μg/ml) were added to triplicate wells. PBMCs cultured without peptides were regarded as the negative controls. Plates were scanned by Elispot Reader System (Cell Technology Inc., Columbia, MD), and the results were analyzed with Elispot software (AID, Strassberg, Germany).

### TCR Sequencing

DNA extracted from PBMCs was prepared for TCR β-chain amplification using Short Read iR-Profile Reagent System HTBI-vc and sequenced using the NextSeq system. V-D-J gene segments in CDR3 sequences were identified by MiXCR (v2.1.10). Basic quantification of clonotypes was assessed with VDJ tools (v1.1.10). High-frequency clones were defined as T-cell clones with a frequency of ≥10^−3^. TCRs from simulated PBMCs were further analyzed by comparing the frequency of each T-cell clone being stimulated by the mutant peptide with the same clone being stimulated by the wild-type peptide. Neoantigen-specific T-cell clones were identified with an odd ratio higher than 10 and a value of *p* < 0.01.

### Statistics

All the statistical analyses were conducted by GraphPad Prism version 8.0 (GraphPad Software, USA). TCR sequencing was compared using a one-sided Fisher's exact test. Other values were compared using an unpaired two-tailed Student's *t*-test. A value of *p* < 0.05 was considered statistically significant.

## Data Availability Statement

The data in this study is available on the GEO - GSE150972. Other raw data supporting the conclusions of this article will be made available by the authors, without undue reservation, to any qualified researcher.

## Ethics Statement

This study was approved by the Institutional Review Board of Tongji Medical College of Huazhong University of Science and Technology. The patient gave his written informed consent for the collection of blood and tissue samples in accordance with the Declaration of Helsinki. Written informed consent was obtained from the patient for the publication of any potentially identifiable images or data included in this article.

## Author Contributions

DW and YaL designed and performed the experiments. YiL, QY, and JW performed and analyzed the sequencing data. CT, ZZ, YX, YZ, and XL performed data analysis and statistical oversight. FG, YuL, and KZ provided technical assistance. LL and YH were responsible for the provision of study resources, materials, and patient access. DW wrote the manuscript. All authors read and approved the final manuscript.

## Conflict of Interest

XL, YiL, ZZ, and YX were employed by the company YuceBio Technology Co. The remaining authors declare that the research was conducted in the absence of any commercial or financial relationships that could be construed as a potential conflict of interest.

## References

[1] Siegel RL Miller KD Jemal A Cancer statistics, 2019. CA Cancer J Clin. (2019) 69:7–34. 10.3322/caac.21551 30620402

[2] Mok TS Wu Y-L Thongprasert S Yang C-H Chu D-T Saijo N Gefitinib or carboplatin–paclitaxel in pulmonary adenocarcinoma. N Engl J Med. (2009) 361:947–57. 10.1056/NEJMoa0810699 19692680

[3] Kwak EL Bang Y-J Camidge DR Shaw AT Solomon B Maki RG. Anaplastic lymphoma kinase inhibition in non–small-cell lung cancer. N Engl J Med. (2010) 363:1693–703. 10.1056/NEJMoa1006448 20979469 PMC3014291

[4] Yatabe Y Kerr KM Utomo A Rajadurai P Du X Chou T-Y. EGFR mutation testing practices within the Asia Pacific region: results of a multicenter diagnostic survey. J Thorac Oncol. (2015) 10:438–45. 10.1097/JTO.0000000000000422 25376513 PMC4342317

[5] Tan CS Gilligan D Pacey S. Treatment approaches for EGFR-inhibitor-resistant patients with non-small-cell lung cancer. Lancet Oncol. (2015) 16:e447–59. 10.1016/S1470-2045(15)00246-6 26370354

[6] Sullivan I Planchard D. Osimertinib in the treatment of patients with epidermal growth factor receptor T790M mutation-positive metastatic non-small cell lung cancer: clinical trial evidence and experience. Ther Adv Respir Dis. (2016) 10:549–65. 10.1177/1753465816670498 27784815 PMC5933598

[7] Yang Z Yang N Ou Q Xiang Y Jiang T Wu X. Investigating novel resistance mechanisms to third-generation EGFR tyrosine kinase inhibitor osimertinib in non–small cell lung cancer patients. Clin Cancer Res. (2018) 24:3097–107. 10.1158/1078-0432.CCR-17-2310 29506987

[8] Borghaei H Paz-Ares L Horn L Spigel DR Steins M Ready NE. Nivolumab versus docetaxel in advanced nonsquamous non–small-cell lung cancer. N Engl J Med. (2015) 373:1627–39. 10.1056/NEJMoa1507643 26412456 PMC5705936

[9] Reck M Rodríguez-Abreu D Robinson AG Hui R Csoszi T Fülöp A. Pembrolizumab versus chemotherapy for PD-L1–positive non–small-cell lung cancer. N Engl J Med. (2016) 375:1823–33. 10.1056/NEJMoa1606774 27718847

[10] Gainor JF Shaw AT Sequist LV Fu X Azzoli CG Piotrowska Z. EGFR mutations and ALK rearrangements are associated with low response rates to PD-1 pathway blockade in non–small cell lung cancer: a retrospective analysis. Clin Cancer Res. (2016) 22:4585–93. 10.1158/1078-0432.CCR-15-3101 27225694 PMC5026567

[11] Lee CK Man J Lord S Links M Gebski V Mok T. Checkpoint inhibitors in metastatic EGFR-mutated non–small cell lung cancer—a meta-analysis. J Thorac Oncol. (2017) 12:403–7. 10.1016/j.jtho.2016.10.007 27765535

[12] Kotake M Murakami H Kenmotsu H Naito T Takahashi T. High incidence of interstitial lung disease following practical use of osimertinib in patients who had undergone immediate prior nivolumab therapy. Ann Oncol. (2017) 28:669–70. 10.1093/annonc/mdw647 27993813

[13] Schumacher TN Schreiber RD Neoantigens in cancer immunotherapy. Science. (2015) 348:69–74. 10.1126/science.aaa4971 25838375

[14] Rizvi NA Hellmann MD Snyder A Kvistborg P Makarov V Havel JJ. Mutational landscape determines sensitivity to PD-1 blockade in non–small cell lung cancer. Science. (2015) 348:124–8. 10.1126/science.aaa1348 25765070 PMC4993154

[15] Ott PA Dotti G Yee C Goff SL. An update on adoptive T-cell therapy and neoantigen vaccines. Am Soc Clin Oncol Educ Book. (2019) 39:e70–8. 10.1200/EDBK_238001 31099621

[16] Keskin DB Anandappa AJ Sun J Tirosh I Mathewson ND Li S. Neoantigen vaccine generates intratumoral T cell responses in phase Ib glioblastoma trial. Nature. (2019) 565:234–9. 10.1038/s41586-018-0792-9 30568305 PMC6546179

[17] Jiang T Shi T Zhang H Hu J Song Y Wei J. Tumor neoantigens: from basic research to clinical applications. J Hematol Oncol. (2019) 12:93. 10.1186/s13045-019-0787-5 31492199 PMC6731555

[18] Yi M Qin S Zhao W Yu S Chu Q Wu K. The role of neoantigen in immune checkpoint blockade therapy. Exp Hematol Oncol. (2018) 7:28. 10.1186/s40164-018-0120-y 30473928 PMC6240277

[19] Ofuji K Tada Y Yoshikawa T Shimomura M Yoshimura M Saito K. A peptide antigen derived from EGFR T790M is immunogenic in non-small cell lung cancer. Int J Oncol. (2015) 46:497–504. 10.3892/ijo.2014.2787 25532027 PMC4277252

[20] Roudko V Greenbaum B Bhardwaj N. Computational prediction and validation of tumor-associated neoantigens. Front Immunol. (2020) 11:27. 10.3389/fimmu.2020.00027 32117226 PMC7025577

[21] McGranahan N Furness AJ Rosenthal R Ramskov S Lyngaa R Saini SK. Clonal neoantigens elicit T cell immunoreactivity and sensitivity to immune checkpoint blockade. Science. (2016) 351:1463–9. 10.1126/science.aaf1490 26940869 PMC4984254

[22] Yu S Sha H Qin X Chen Y Li X Shi M. EGFR E746-A750 deletion in lung cancer represses antitumor immunity through the exosome-mediated inhibition of dendritic cells. Oncogene. (2020) 39:2643–57. 10.1038/s41388-020-1182-y 32001818

[23] Jia Y Li X Jiang T Zhao S Zhao C Zhang L. EGFR-targeted therapy alters the tumor microenvironment in EGFR-driven lung tumors: implications for combination therapies. Int J Cancer. (2019) 145:1432–44. 10.1002/ijc.32191 30784054

[24] Jia Y Zhao S Jiang T Li X Zhao C Liu Y. Impact of EGFR-TKIs combined with PD-L1 antibody on the lung tissue of EGFR-driven tumor-bearing mice. Lung Cancer. (2019) 137:85–93. 10.1016/j.lungcan.2019.09.016 31563735

[25] Peng S Wang R Zhang X Ma Y Zhong L Li K. EGFR-TKI resistance promotes immune escape in lung cancer via increased PD-L1 expression. Mol Cancer. (2019) 18:165. 10.1186/s12943-019-1073-4 31747941 PMC6864970

[26] Melosky B Chu Q Juergens RA Leighl N Ionescu D Tsao MS. Breaking the biomarker code: PD-L1 expression and checkpoint inhibition in advanced NSCLC. Cancer Treat Rev. (2018) 65:65–77. 10.1016/j.ctrv.2018.02.005 29567557

[27] Sugiyama E Togashi Y Takeuchi Y Shinya S Tada Y Kataoka K. Blockade of EGFR improves responsiveness to PD-1 blockade in EGFR-mutated non–small cell lung cancer. Sci Immunol. (2020) 5:aav3937. 10.1126/sciimmunol.aav3937 32005679

[28] Ji M Liu Y Li Q Li X Ning Z Zhao W. PD-1/PD-L1 expression in non-small-cell lung cancer and its correlation with EGFR/KRAS mutations. Cancer Biol Ther. (2016) 17:407–13. 10.1080/15384047.2016.1156256 26954523 PMC4910919

[29] Garassino MC Cho B-C Kim J-H Mazières J Vansteenkiste J Lena H. Durvalumab as third-line or later treatment for advanced non-small-cell lung cancer (ATLANTIC): an open-label, single-arm, phase 2 study. Lancet Oncol. (2018) 19:521–36. 10.1016/S1470-2045(18)30144-X 29545095 PMC7771363

[30] Lisberg A Cummings A Goldman JW Bornazyan K Reese N Wang T. A phase II study of pembrolizumab in EGFR-mutant, PD-L1+, tyrosine kinase inhibitor naïve patients with advanced NSCLC. J Thorac Oncol. (2018) 13:1138–45. 10.1016/j.jtho.2018.03.035 29874546 PMC6063769

[31] Goodman AM Kato S Bazhenova L Patel SP Frampton GM Miller V. Tumor mutational burden as an independent predictor of response to immunotherapy in diverse cancers. Mol Cancer Ther. (2017) 16:2598–608. 10.1158/1535-7163.MCT-17-0386 28835386 PMC5670009

[32] Le DT Durham JN Smith KN Wang H Bartlett BR Aulakh LK. Mismatch repair deficiency predicts response of solid tumors to PD-1 blockade. Science. (2017) 357:409–13. 10.1126/science.aan6733 28596308 PMC5576142

[33] Smith KN Llosa NJ Cottrell TR Siegel N Fan H Suri P Persistent mutant oncogene specific T cells in two patients benefitting from anti-PD-1. J Immunother Cancer. (2019) 7:40 10.1186/s40425-018-0492-x 30744692 PMC6371497

[34] Asadi-Ghalehni M Ghaemmaghami M Klimka A Javanmardi M Navari M Rasaee MJ. Cancer immunotherapy by a recombinant phage vaccine displaying EGFR mimotope: an *in vivo* study. Immunopharmacol Immunotoxicol. (2015) 37:274–9. 10.3109/08923973.2015.1027917 25990849

[35] Malekzadeh P Pasetto A Robbins PF Parkhurst MR Paria BC Jia L. Neoantigen screening identifies broad TP53 mutant immunogenicity in patients with epithelial cancers. J Clin Invest. (2019) 129:1109–14. 10.1172/JCI123791 30714987 PMC6391139

[36] Anagnostou V Smith KN Forde PM Niknafs N Bhattacharya R White J. Evolution of neoantigen landscape during immune checkpoint blockade in non–small cell lung cancer. Cancer Discov. (2017) 7:264–76. 10.1158/1538-7445.AM2017-NG01 28031159 PMC5733805

[37] Consortium APG. AACR Project GENIE: powering precision medicine through an international consortium. Cancer Discov. (2017) 7:818–31. 10.1158/2159-8290.CD-17-0151 28572459 PMC5611790

[38] Cafri G Yossef R Pasetto A Deniger DC Lu YC Parkhurst M. Memory T cells targeting oncogenic mutations detected in peripheral blood of epithelial cancer patients. Nat Commun. (2019) 10:449. 10.1038/s41467-019-08304-z 30683863 PMC6347629

[39] Fehlings M Jhunjhunwala S Kowanetz M O'Gorman WE Hegde PS Sumatoh H. Late-differentiated effector neoantigen-specific CD8+ T cells are enriched in peripheral blood of non-small cell lung carcinoma patients responding to atezolizumab treatment. J Immunother Cancer. (2019) 7:249. 10.1186/s40425-019-0695-9 31511069 PMC6740011

[40] Gros A Tran E Parkhurst MR Ilyas S Pasetto A Groh EM. Recognition of human gastrointestinal cancer neoantigens by circulating PD-1+ lymphocytes. J Clin Invest. (2019) 129:4992–5004. 10.1172/JCI127967 31609250 PMC6819109

[41] Liu YY Yang QF Yang JS Cao RB Liang JY Liu YT. Characteristics and prognostic significance of profiling the peripheral blood T-cell receptor repertoire in patients with advanced lung cancer. Int J Cancer. (2019) 145:1423–31. 10.1002/ijc.32145 30664810

[42] Yost KE Satpathy AT Wells DK Qi Y Wang C Kageyama R. Clonal replacement of tumor-specific T cells following PD-1 blockade. Nat Med. (2019) 25:1251–9. 10.1038/s41591-019-0522-3 31359002 PMC6689255

[43] Pan D Zhou D Cai W Wu W Tan WL Zhou C. Immunogenicity of Del19 EGFR mutations in Chinese patients affected by lung adenocarcinoma. BMC Immunol. (2019) 20:43. 10.1186/s12865-019-0320-1 31722672 PMC6854806

[44] Gonzalez-Galarza FF Christmas S Middleton D Jones AR. Allele frequency net: a database and online repository for immune gene frequencies in worldwide populations. Nucleic Acids Res. (2011) 39:D913–9. 10.1093/nar/gkq1128 21062830 PMC3013710

[45] Alspach E Lussier DM Miceli AP Kizhvatov I DuPage M Luoma AM. MHC-II neoantigens shape tumour immunity and response to immunotherapy. Nature. (2019) 574:696–701. 10.1038/s41586-019-1671-8 31645760 PMC6858572

[46] Koboldt DC Larson DE Wilson RK. Using VarScan 2 for germline variant calling and somatic mutation detection. Curr Protoc Bioinform. (2013) 44:15–4. 10.1002/0471250953.bi1504s44 25553206 PMC4278659

[47] Raine KM Van Loo P Wedge DC Jones D Menzies A Butler AP. ascatNgs: Identifying somatically acquired copy-number alterations from whole-genome sequencing data. Curr Protoc Bioinform. (2016) 56:15–9. 10.1002/cpbi.17 27930809 PMC6097604

[48] Shukla SA Rooney MS Rajasagi M Tiao G Dixon PM Lawrence MS. Comprehensive analysis of cancer-associated somatic mutations in class I HLA genes. Nat Biotechnol. (2015) 33:1152–8. 10.1038/nbt.3344 26372948 PMC4747795

[49] Hoof I Peters B Sidney J Pedersen LE Sette A Lund O. NetMHCpan, a method for MHC class I binding prediction beyond humans. Immunogenetics. (2009) 61:1–13. 10.1007/s00251-008-0341-z 19002680 PMC3319061

[50] Danilova L Anagnostou V Caushi JX Sidhom JW Guo H Chan HY. The mutation-associated neoantigen functional expansion of specific T cells (MANAFEST) assay: a sensitive platform for monitoring antitumor immunity. Cancer Immunol Res. (2018) 6:888–99. 10.1158/2326-6066.CIR-18-0129 29895573 PMC6072595

[51] Su S Hu B Shao J Shen B Du J Du Y. CRISPR-Cas9 mediated efficient PD-1 disruption on human primary T cells from cancer patients. Sci Rep. (2016) 6:20070. 10.1038/srep20070 26818188 PMC4730182

