# Supplementary figures and images for: Identification of Clonal Neoantigens Derived From Driver Mutations in an EGFR-Mutated Lung Cancer Patient Benefitting From Anti-PD-1
Source: Front Immunol. 2020 Jul 23;11:1366. doi: 10.3389/fimmu.2020.01366 (PMC7390822; doi:10.3389/fimmu.2020.01366)

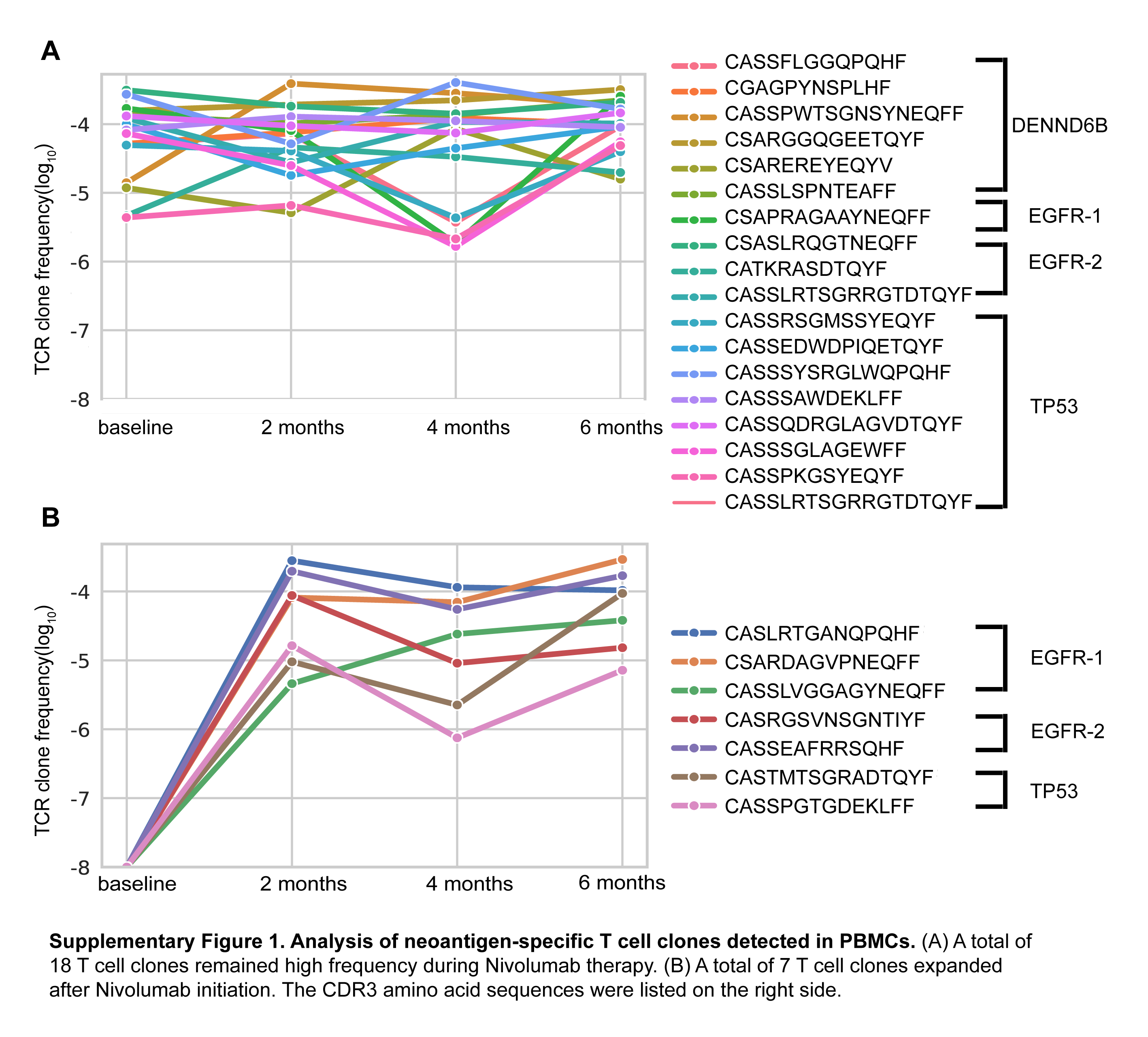

Supplement: Supplementary file 3 [file Image_1.TIF]
